# Impact of the COVID‐19 related border restrictions on influenza and other common respiratory viral infections in New Zealand

**DOI:** 10.1111/irv.13247

**Published:** 2024-02-13

**Authors:** Q. Sue Huang, Nikki Turner, Tim Wood, Andrew Anglemyer, Peter McIntyre, Nayyereh Aminisani, Tony Dowell, Adrian Trenholme, Cass Byrnes, Michelle Balm, Christine McIntosh, Sarah Jefferies, Cameron C. Grant, Annette Nesdale, Hazel C. Dobinson, Priscilla Campbell‐Stokes, Karen Daniells, Jemma Geoghegan, Joep de Ligt, Lauren Jelley, Ruth Seeds, Tineke Jennings, Megan Rensburg, Jort Cueto, Ernest Caballero, Joshma John, Emmanuel Penghulan, Chor Ee Tan, Xiaoyun Ren, Klarysse Berquist, Meaghan O'Neill, Maritza Marull, Chang Yu, Andrea McNeill, Tomasz Kiedrzynski, Sally Roberts, Colin McArthur, Alicia Stanley, Susan Taylor, Conroy Wong, Shirley Lawrence, Michael G. Baker, Amanda Kvalsvig, Koen Van Der Werff, Gary McAuliffe, Hanna Antoszewska, Meik Dilcher, Jennifer Fahey, Anja Werno, Juliet Elvy, Jenny Grant, Michael Addidle, Nicolas Zacchi, Chris Mansell, Marc‐Alain Widdowson, Paul G. Thomas, BorderRestrictionImpactOnFluRSV Consortium, Richard J. Webby

**Affiliations:** ^1^ Institute of Environmental Science and Research Wellington New Zealand; ^2^ University of Auckland Auckland New Zealand; ^3^ University of Otago Dunedin New Zealand; ^4^ Te Whatu Ora, Health New Zealand Counties Manukau Auckland New Zealand; ^5^ Te Whatu Ora, Health New Zealand Te Toka Tumai Auckland Auckland New Zealand; ^6^ Te Whatu Ora, Health New Zealand Capital, Coast and Hutt Valley Wellington New Zealand; ^7^ Regional Public Health Te Whatu Ora, Health New Zealand Capital, Coast and Hutt Valley Wellington New Zealand; ^8^ Te Pou Hauora Tūmatanui, the Public Health Agency Manatū Hauora, Ministry of Health Wellington New Zealand; ^9^ Te Whatu Ora, Health New Zealand Waitaha Canterbury Christchurch New Zealand; ^10^ Southern Community Laboratories Dunedin New Zealand; ^11^ Te Whatu Ora, Health New Zealand Hauora a Toi Bay of Plenty Tauranga New Zealand; ^12^ Te Whatu Ora, Health New Zealand Waikato Hamilton New Zealand; ^13^ Institute of Tropical Medicine Antwerp Belgium; ^14^ WHO Collaborating Centre St Jude Children's Research Hospital Memphis Tennessee USA

**Keywords:** acute respiratory illness, common respiratory viral infections, influenza infection, public health and social measures, respiratory syncytial viral infection, severe acute respiratory infections

## Abstract

**Background:**

New Zealand's (NZ) complete absence of community transmission of influenza and respiratory syncytial virus (RSV) after May 2020, likely due to COVID‐19 elimination measures, provided a rare opportunity to assess the impact of border restrictions on common respiratory viral infections over the ensuing 2 years.

**Methods:**

We collected the data from multiple surveillance systems, including hospital‐based severe acute respiratory infection surveillance, SHIVERS‐II, ‐III and ‐IV community cohorts for acute respiratory infection (ARI) surveillance, HealthStat sentinel general practice (GP) based influenza‐like illness surveillance and SHIVERS‐V sentinel GP‐based ARI surveillance, SHIVERS‐V traveller ARI surveillance and laboratory‐based surveillance. We described the data on influenza, RSV and other respiratory viral infections in NZ before, during and after various stages of the COVID related border restrictions.

**Results:**

We observed that border closure to most people, and mandatory government‐managed isolation and quarantine on arrival for those allowed to enter, appeared to be effective in keeping influenza and RSV infections out of the NZ community. Border restrictions did not affect community transmission of other respiratory viruses such as rhinovirus and parainfluenza virus type‐1. Partial border relaxations through quarantine‐free travel with Australia and other countries were quickly followed by importation of RSV in 2021 and influenza in 2022.

**Conclusion:**

Our findings inform future pandemic preparedness and strategies to model and manage the impact of influenza and other respiratory viral threats.

## 1 INTRODUCTION

COVID‐19, declared as a public health emergency of international concern by the World Health Organization (WHO) on 30 January 2020, was first identified in New Zealand (NZ) on 28 February 2020. From 19 March 2020, NZ responded to the COVID‐19 pandemic with stringent public health and social measures (PHSMs) including border restrictions and a national lockdown that included strict stay‐at‐home orders at Alert Level 4 with a range of mandated actions and restrictions such as closure of all public and education facilities including early childhood education centres and schools.^1^, ^2^ These measures were successful in containing the first wave of the COVID‐19 outbreak with elimination of community transmission for 101 consecutive days from 1 May to 10 August 2020.^3^, ^4^ We previously reported that no community transmission of influenza and respiratory syncytial virus (RSV) was identified from May 2020.^5^

Prior to 2020, the impacts of border closures on disease spread were largely unknown, and their use as a pandemic policy was advised against by WHO,^6^ in part due to their potential to be discriminatory and worsen economic and social disruption. However, in response to the COVID‐19 pandemic, nearly every country introduced international border closures with varying durations and stringency.^7^

**FIGURE 1:**
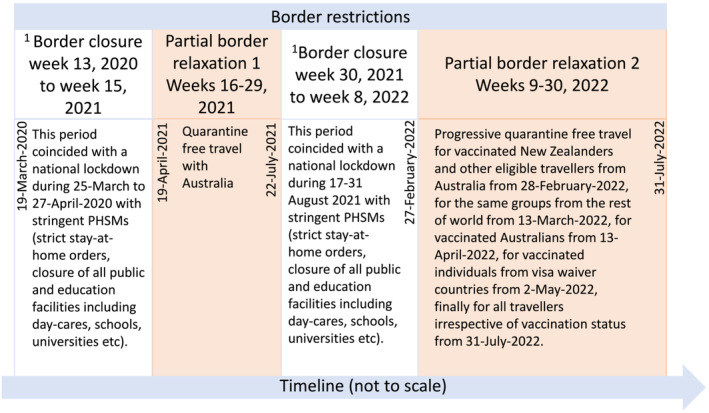
Timeline of New Zealand's border restrictions. ^1^Border closure = borders close to all but New Zealand citizens and permanent residents. For those allowed to enter, they are required to comply with mandatory government‐managed isolation and quarantine (MIQ) in designated facilities on arrival. PHSMs, public health and social measures.

NZ implemented a range of border restrictions for more than 2 years from 19 March 2020 to 31 July 2022 (Figure 1) with the intention of preventing imported COVID‐19 cases from establishing community transmission in the country. Initially, restrictions included mandatory government‐managed isolation and quarantine (MIQ) in designated facilities on arrival for all people seeking to enter the country. After more than 12 months of border closure, partial border relaxation was introduced for around 3 months (from 19 April 2021) allowing quarantine‐free travel with Australia but reinstated from 23 July 2021 until February 2022 with progressive relaxation thereafter. Quarantine‐free travel was permitted for vaccinated New Zealanders and other eligible travellers from Australia from 28 Feburary 2022,^8^ for vaccinated Australians from 13 April 2022, for vaccinated travellers from NZ's list of 60 visa‐waiver countries from 2 May 2022, and finally, for all travellers irrespective of vaccination status from 31 July 2022.

There are some data on the impact of border closures on influenza during the COVID‐19 pandemic from studies conducted in China,^9^, ^10^ Hong Kong,^11^ Taiwan,^12^ Singapore^13^ and Australia.^14^ However, these studies only evaluated the overall effect of a set of combined PHSMs, without disentangling the specific effects of individual PHSM.^15^ NZ has a unique situation where stringent border restrictions remained in place for 2 years, while in‐country stringent PHSMs were largely absent. A 3‐month border opening with Australia during broader border closure created opportunities for importation of viruses from Australia. This allowed us to distinguish the effect of the stringent border restrictions from other stringent measures (stay‐at‐home orders, school closure etc.) on influenza, RSV and other respiratory viral infections.

Over the last 10 years, NZ has invested in comprehensive respiratory virus surveillance platforms including patients admitted acutely to hospitals, those making consultation visits to general practitioners (GPs) and community cohorts with non‐medically attended acute respiratory infections. During the COVID‐19 pandemic, most of these surveillance programmes remained largely intact and enhanced with additional research. This provided a rare ‘real‐world’ quality dataset to examine temporal associations of border restrictions and importations of respiratory viruses with varying disease severity and then their in‐country epidemiology in an island nation. Understanding the effect of border restrictions on these viral infections and associated diseases is critical to informing pandemic influenza preparedness and planning countermeasures for seasonal influenza, RSV and other respiratory viral infections.

Here, we describe data collected from multiple surveillance systems on influenza, RSV and other respiratory viral infections in NZ before, during and after various stages of the COVID‐19 related border restrictions.

## 2 METHODS

### 2.1 Hospital‐based severe acute respiratory infection (SARI) surveillance

The population‐based hospital SARI surveillance among residents (catchment population of one million people across the central, east and south Auckland region) was established in 2012 as the first iteration of the Southern Hemisphere Influenza and Vaccine Effectiveness Research and Surveillance (SHIVERS‐I) study.^16^, ^17^ Active surveillance periods for hospital intensive care units (ICUs) were year‐round, and for general medical/paediatrics wards, usually from May to September of each year but started from 7 February 2022 due to COVID‐19 community transmission. Research nurses reviewed daily records of all overnight general medical/paediatrics wards and ICU admitted acute inpatients to identify any with suspected acute respiratory illnesses. They enrolled those patients with cough and history of fever (subjective fever or measured temperature ≥38°C) and onset within the past 10 days, as defined by the WHO as SARI, and collected a nasopharyngeal or nasal or throat swab.

### 2.2 SHIVERS‐II, ‐III and ‐IV community cohorts for acute respiratory infection (ARI) surveillance

SHIVERS‐II, ‐III and ‐IV are three prospective, longitudinal (7 years), community cohorts in Wellington.^5^ SHIVERS‐II is an adult cohort operating since 2018 with approximately 1400 participants in 2020, 1100 in 2021 and 900 in 2022. SHIVERS‐III is an infant cohort operating since 2019 with approximately 80 participants in 2020, 300 in 2021 and 600 in 2022. SHIVERS‐IV is a household cohort operating since 2021 with around 500 families (approximately 1000 household members in 2021 and 1700 in 2022).

Each year, the active surveillance period for the three cohorts typically occurs from May to September. In 2022, surveillance started on 7 February due to COVID‐19 community transmission. The study staff sent weekly surveys to participants regarding their respiratory symptoms. Nurses reviewed participant's symptom reports and identified those meeting relevant case definitions: ARI—‘an acute respiratory illness with fever or feverishness and/or one of following symptoms (cough, running nose, wheezing, sore throat, shortness of breath and loss of sense of smell/taste) with onset in the past 10 days’; influenza‐like illness (ILI)—‘acute respiratory illness with cough and fever/measured fever of ≥38°C and onset within the past 10 days’. Nurses guided those with ARI/ILI to take a nasopharyngeal or nasal swab.

### 2.3 HealthStat's sentinel general practice (GP)‐based ILI surveillance and SHIVERS‐V sentinel GP‐based ARI surveillance

HealthStat GP‐based ILI surveillance consists of a nationally representative random sample of approximately 300 sentinel GPs.^5^, ^18^ The case definition for ILI: ‘an acute upper respiratory tract infection, with abrupt onset of two or more symptoms from chills, fever, headache and myalgia’. This surveillance monitored the number of people who consult GPs with ILI and collected automated weekly extracts of ILI read codes from practice management systems.^19^ This surveillance did not include virological surveillance.

SHIVERS‐V sentinel GP‐based ARI surveillance (from eight sentinel GPs in Auckland, Wellington and Dunedin) was established in the middle of June 2021. If a consultation seeking patient met the ARI case definition (the same as SHIVERS‐II, ‐III and ‐IV ARI), a nasopharyngeal or nasal swab was collected.

### 2.4 SHIVERS‐V traveller ARI surveillance

SHIVERS‐V traveller ARI surveillance was established on 10 May 2021 and was operational until 27 February 2022. All travellers staying in 32 MIQ facilities were required to test for severe acute respiratory syndrome coronavirus 2 (SARS‐CoV‐2). This surveillance included five hospital‐based laboratories covering 29 MIQ facilities. A daily electronic extract from the COVID‐19 éclair (https://www.sysmex-ap.com/product/eclair/) database was generated for each participating laboratory to identify any traveller with a suspected acute respiratory infection who met the ARI case definition (the same as SHIVERS‐V GP ARI). If there was any left‐over specimen after the SARS‐CoV‐2 testing, the specimen was tested for other respiratory viruses.

### 2.5 Laboratory‐based surveillance

The laboratory‐based surveillance for influenza, RSV and other common respiratory viruses is carried out year‐round by the NZ virus laboratory network consisting of the National Influenza Centre at ESR (Institute of Environmental Science and Research) and six hospital laboratories in Auckland (two hospitals), Waikato, Wellington, Christchurch and Dunedin. This laboratory network tests specimens ordered by clinicians for hospital inpatients and outpatients during normal clinical practice (serving approximately 70% of the NZ population). Sample collection is based on clinician judgement.

Additionally, this network conducts testing for public health surveillance including hospital‐based SARI, GP‐based ILI/ARI, and SHIVERS‐II, ‐III, ‐IV and ‐V ILI/ARI surveillance. The collected nasopharyngeal or nasal swabs were tested by polymerase chain reactions (PCRs)^17^ specifically for influenza virus, RSV, rhinovirus, parainfluenza virus types 1–3, enterovirus, adenovirus, human metapneumovirus and SARS‐CoV‐2.^20^

### 2.6 Data analyses

Study data were captured using REDCap 10.0.19 electronic data capture tools.^21^ Analyses were performed in Stata 16.1 (StataCorp LLC).

The observed incidence rates of influenza/RSV/Rhinovirus‐PCR‐confirmed SARI or ARI or ILI were corrected each week to account for missed swabs from ARI cases by applying the influenza/RSV/Rhinovirus positivity rate of those tested to those not tested (corrected number of influenza/RSV/Rhinovirus‐PCR‐confirmed SARI or ILI or ARI events = number of SARI or ILI or ARI × actual number of influenza/RSV/Rhinovirus‐PCR‐confirmed SARI or ILI or ARI ÷ actual number of SARI or ILI or ARI swabs).

Based on SARI and ILI surveillance data from 2015–2019, the start of the annual influenza season and intensity level of the influenza epidemics was defined by using the moving epidemic method.^18^, ^22^, ^23^

Laboratory‐based surveillance data used the median of the annual total of the specified week period over the years 2015–2019 to represent the reference period for that week period. Median and interquartile ranges were calculated for the number of viruses reported during 2015–2019; Percentage change = (no. virus − median no. virus [2015–2019]) ÷ median no. virus (2015–2019) × 100.

The 95% confidence intervals (CIs) for proportions (incidence rates) were calculated using the binomial distribution.

### 2.7 Ethics statement

The NZ Northern A Health and Disability Ethics Committee approved the SHIVERS‐I, ‐II, ‐III, ‐IV and ‐V studies (NTX/11/11/102). The GP‐based ARI/ILI and laboratory‐based surveillance are conducted in accordance with the Public Health Act, and thus, ethics approval was not required.

## 3 RESULTS

**FIGURE 2:**
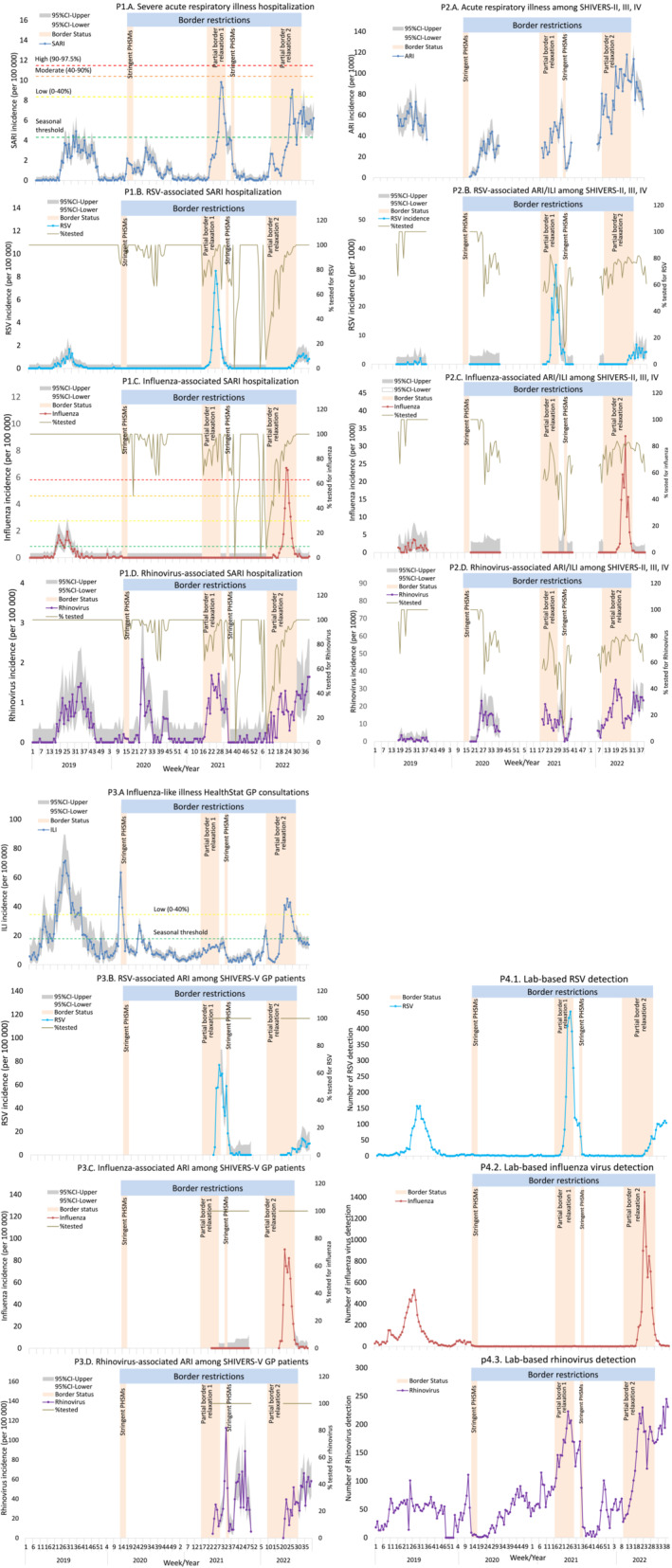
Temporal distribution of acute respiratory infections (ARIs) and associated influenza, RSV, and rhinovirus detections with (2020–2022) and without (2019) border restrictions. Panel 1: P1.A. Hospital‐based severe acute respiratory infection incidence rate, P1.B. RSV ‐associated SARI, P1.C. Influenza ‐associated SARI, P1.D. Rhinovirus‐associated SARI. Panel 2: P2.A. SHIVERS‐II, ‐III and ‐IV cohort‐based ARI incidence rate, P2.B. RSV‐associated ARI/ILI, P2.C. Influenza‐associated ARI/ILI, P2.D. Rhinovirus‐associated ARI/ILI. Panel 3: P3.A. ILI consultations among HealthStat GP patients, P3.B. RSV‐associated ARI among SHIVERS‐V GP patients, P3.C. Influenza‐associated ARI among SHIVERS‐V GP patients, P3.D. Rhinovirus‐associated ARI among SHIVERS‐V GP patients. Panel 4: P4.1. Lab‐based RSV, P4.2. Lab‐based influenza, P4.3. Lab‐based rhinovirus detection. GP, general practice; ILI, influenza‐like illness; PHSMs, public health and social measures; SARI, severe acute respiratory infection; SHIVERS‐II, ‐III, ‐IV and –V, the 2nd, 3rd, 4th, 5th iterations of the southern hemisphere influenza and vaccine effectiveness research and surveillance programme. The calculation for epidemic threshold and influenza activity are described in the Section 2. A patient with cough and history of fever (subjective fever or measured temperature ≥38°C) and onset within the past 10 days meets the SARI case definition if hospitalised or meets the ILI case definition if participating in the SHIVERS‐II and ‐III study during 2019. The ARI case definition among SHIVERS‐II, ‐III, ‐IV and ‐V participants refers to an “acute respiratory illness with fever or feverishness and/or one of following symptoms (cough, runny nose, wheezing, sore throat, shortness of breath, loss of sense of smell/taste) with onset in the past 10 days”. Partial border relaxation 1 refers to brief introduction of quarantine‐free travel with Australia during 19 April 2021 to 22 July 2021. Partial border relaxation 2 refers to progressive border relaxation between 28 Feburary 2022 to 31 July 2022. Introduction of quarantine‐free travel initially for vaccinated New Zealanders from Australia on 28 Feburary 2022 and for the same groups from the rest of the world on 13 March 2022, then for vaccinated Australians from 13 April 2022 and vaccinated travellers from NZ's visa‐waiver countries from 2 May 2022 onwards.

We have previously reported complete absence of community transmission of influenza and RSV after May 2020.^5^ While this absence continued during border closure, multiple surveillance systems consistently showed that re‐introduction of RSV and influenza into the NZ community were temporally associated with partial border relaxations in 2021 and 2022, respectively (Figure 2).

Hospital‐based surveillance recorded low SARI incidence rates in 2020, all below the seasonal threshold defined by the reference period of 2015–2019 (Figure 2P1.A.). In 2021, RSV‐associated SARI hospitalisation rates (Figure 2P1.B.) were first reported 2 weeks following commencement of quarantine‐free travel with Australia where inter‐seasonal RSV outbreaks and very low influenza and COVID‐19 activity were reported.^24^, ^25^ In 2022, influenza‐associated SARI hospitalisation rates (Figure 2P1.C.) were first reported 5 weeks after 28 February partial border relaxation. Rhinovirus‐associated SARI hospitalisation rates (Figure 2P1.D.) were reported consistently throughout 2019–2022 regardless of border restrictions. Overall, proportion of swabs tested among SARI cases remained on average at 87% during border restrictions (between Week 13 of 2020 to Week 30 of 2022; Table S1).

SHIVERS‐II, ‐III and ‐IV community cohort surveillance results, consistent with the patterns detected by hospital‐based SARI surveillance, showed that ARI incidence rates (Figure 2P2.A.) were mainly driven by RSV (Figure 2P2.B.), which peaked in late June 2021, and influenza (Figure 2P2.C.), which peaked in late June 2022. Rhinovirus‐associated ARI incidence rates (Figure 2P2.D.) were reported throughout 2019–2022. Overall, proportion of swabs tested among ARI/ILI cases remained on average at 72% during border restrictions (Table S1).

The HealthStat sentinel GP‐based ILI rates were mostly below the seasonal threshold during 2020–2021 and at a low level during 2022 (Figure 2P3.A.). SHIVERS‐V sentinel GP‐based ARI surveillance results, like other surveillance streams, detected high incidence rates of RSV in mid‐July 2021 (Figure 2P3.B.), influenza in late June 2022 (Figure 2P3.C.), and rhinovirus throughout 2019–2022 (Figure 2P3.D.). Overall, proportion of swabs tested among ARI cases remained at 100% during border restrictions (Table S1).

The laboratory‐based surveillance detected high numbers of RSV virus in early July 2021 (Figure 2P4.1.), influenza virus in mid‐June 2022 (Figure 2P4.2.) with rhinovirus detections (Figure 2P4.3.) throughout 2019–2022.

**FIGURE 3:**
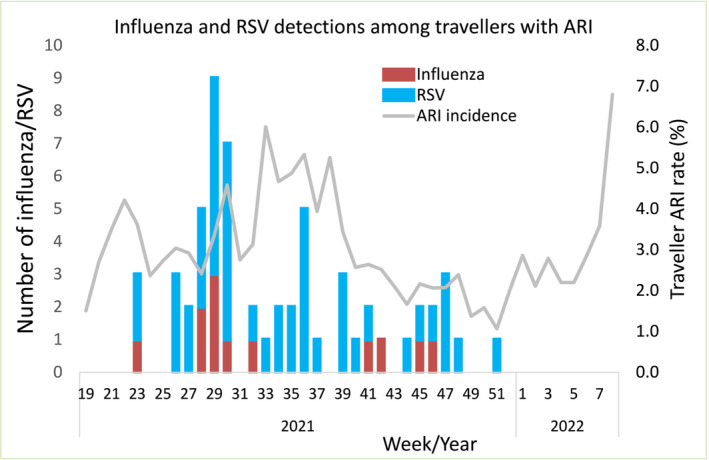
Temporal distribution of influenza and respiratory syncytial virus (RSV) associated acute respiratory infections (ARI) among travellers during 2021–2022. The ARI case definition among travellers refers to an “acute respiratory illness with fever or feverishness and/or one of following symptoms (cough, running nose, wheezing, sore throat, shortness of breath, loss of sense of smell/taste) with onset in the past 10 days.”

SHIVERS‐V travellers' ARI surveillance tested 86,295 samples for SARS‐CoV‐2 from travellers in 29 MIQ facilities. Among travellers with ARI (2484) who had available left‐over samples, 1378 were tested for influenza virus (12 positive) and 1376 were tested for RSV (47 positive; Figure 3). The influenza and RSV cases were scattered throughout border restriction periods.

**FIGURE 4:**
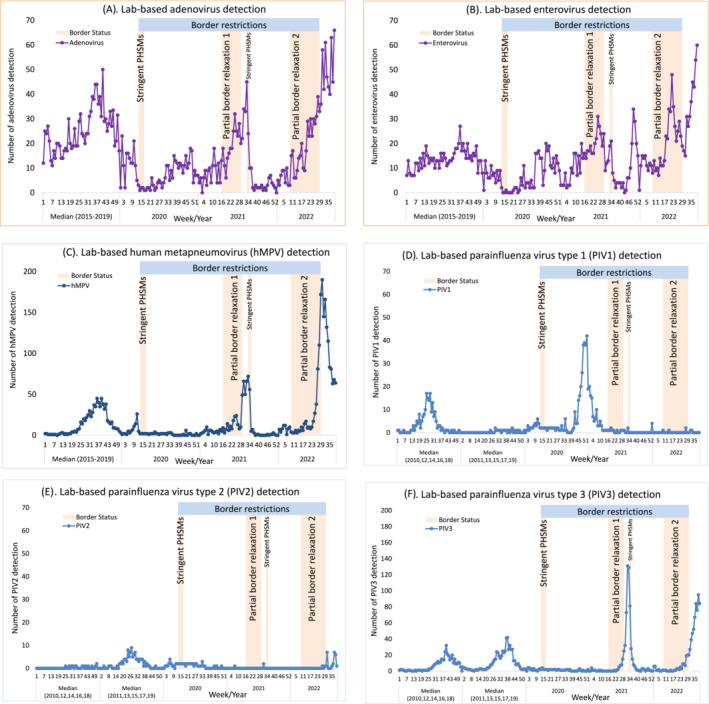
Temporal distribution of other respiratory viral detections during 2020–2022 compared with the reference period of 2015–2019 for adenovirus, enterovirus, and human metapneumovirus (hMPV) or the reference period of even‐numbered* years (2010, 12, 14, 16, 18) or odd‐numbered* years (2011, 13, 15, 17, 19) for parainfluenza virus types 1–3. (A) Lab‐based adenovirus detection. (B) Lab‐based enterovirus detection. (C) Lab‐based human metapneumovirus (hMPV) detection. D Lab‐based parainfluenza virus type 1 (PIV1) detection. (E) Lab‐based parainfluenza virus type 2 (PIV2) detection. (F) Lab‐based parainfluenza virus type 3 (PIV3) detection. (*note: in NZ, PIV1 activity occurred during even‐numbered years while PIV2 activity in odd‐numbered years and PIV3 activity annually. For laboratory‐based PIV1–3 detections during 2003–2022, see supplementary Figure S1).

Table 1 shows the cumulative number of respiratory viruses detected across all surveillance systems and the proportional change for each virus before, during and after border restrictions compared with the reference period of 2015–2019. Between Week 18 of 2020 to Week 8 of 2022, there were 21 influenza virus detections, >99% reduction compared with the reference period. Of these, 17 were from travellers who stayed in MIQ facilities from 21 December 2020 to 27 February 2022, and four were detected from 11 May 2020 to 26 July 2020 with unknown travel information. After border restrictions were relaxed in 2022 (Weeks 9–30), there was a nearly four‐fold increase in influenza virus detections compared to the reference period. Like influenza, marked reductions were also evident for RSV detections (>97%) during border closure (Week 18 of 2020 to Week 15 of 2021). This was followed by a two‐fold increase in RSV detections (Weeks 16–29 of 2021) compared to the reference period soon after quarantine‐free travel with Australia. Other respiratory viruses were less affected by border restrictions. Rhinovirus detections were reduced (82%) during strict lockdown (Weeks 13–17 of 2020), re‐bounded quickly and further increased (18%) during border closure (from Week 18 of 2020 to Week 15 of 2021). Parainfluenza virus type‐1 (PIV1) detections showed a peak from November 2020 to January 2021 despite border closure (Figure 4).

**TABLE 1:** The number of detections of influenza, respiratory syncytial virus (RSV), rhinovirus, human metapneumovirus (hMPV), parainfluenza virus (PIV) types 1–3, enterovirus, and adenovirus and their reduction before, during and after border restrictions compared with the reference period of 2015–2019.

Virus	Border open	^b^Border closure			^d^Partial border relaxation 1	^c^Border closure		^e^Partial border relaxation 2	Border open
	2020	2020	2020	2021	2021	2021	2022	2022	2022
	Weeks 1–12	Weeks 13–17	Weeks 18–52	Weeks 1–15	Weeks 16–29	Weeks 30–52	Weeks 1–8	Weeks 9–30	Weeks 31–39
Influenza									
No.	474	20	6	1	6	6	2	6947	168
Median (2015–19)	206	62	5286	284	1363	2783	126	1826	1994
IQR	90–240	51–139	3349–5311	113–291	763–2660	1928–2819	62–154	980–3322	1407–2419
% Change^a^	130.1	−67.7	−99.9	−99.6	−99.6	−99.8	−98.4	280.4	−91.6
RSV									
No.	34	16	39	13	2286	519	2	223	846
Median (2015–19)	44	85	1829	80	955	893	22	1193	662
IQR	40–45	43–93	1672–2295	62–86	864–1085	833–1376	22–23	1036–1316	603–989
% Change^a^	−22.7	−81.2	−97.9	−83.8	139.4	−41.9	−90.9	−81.3	27.8
Rhinovirus									
No.	518	26	1420	1087	2262	1206	457	2956	1817
Median (2015–19)	232	144	1777	355	750	1130	118	1026	619
IQR	220–303	135–193	1712–1893	285–393	652–828	977–1284	114–152	929–1107	509–628
% Change^a^	123.3	−81.9	−20.1	206.2	201.6	6.7	287.3	188.1	193.5
hMPV									
No.	82	10	50	68	203	334	62	749	917
Median (2015–19)	16	10	714	20	142	557	11	186	326
IQR	12–21	5–13	681–829	18–27	136–164	546–656	7–12	169–208	298–437
% Change^a^	412.5	0.0	−93.0	240.0	43.0	−40.0	463.6	302.7	181.3
PIV									
No.	72	32	379	107	75	531	17	113	597
Median (2015–19)	43	28	808	56	211	627	34	286	299
IQR (2015–19)	38–45	21–37	623–996	53–59	191–238	422–720	33–36	239–287	227–476
% Change^a^	67.4	14.3	−53.1	91.1	−64.5	−15.3	−50.0	−60.5	99.7
Enterovirus									
No.	87	3	274	136	282	249	98	452	343
Median (2015–19)	110	65	504	156	187	325	73	290	167
IQR (2015–19)	105–155	63–73	487–547	142–212	142–191	316–422	66–76	271–302	156–173
% Change^a^	−20.9	−95.4	−45.6	−12.8	50.8	−23.4	34.2	55.9	105.4
Adenovirus									
No.	145	9	276	120	267	222	50	443	465
Median (2015–19)	250	76	1024	308	325	772	162	466	291
IQR (2015–19)	164–256	74–88	762–1154	211–321	300–342	492–865	84–189	452–502	216–412
% Change^a^	−42.0	−88.2	−73.0	−61.0	−17.8	−71.2	−69.1	−4.9	59.8

Abbreviations: hMPV, human matapneumovirus; IQR, interquartile range; PHSMs, public health and social measures; PIV, parainfluenza virus types 1–3; RSV, respiratory synsytial virus.

^a^ % Change = [No. virus − median no. virus (2015–2019)]/median no. virus (2015–2019) × 100.

^b^ Border closure during 19‐March‐2020 to 18‐April‐2021: This period coincided with stringent PHSMs at Alert level 4 during 25‐March to 27‐April‐2020 including strict stay‐at‐home orders and closure of all public and education facilities (early childhood education centres and schools etc).

^c^ Border closure during 23 July 2021 to 27 Feb 2022: this period coincided with a 2‐week stringent PHSMs at Alert level 4 during 17–31 August 2021 including strict stay‐at‐home orders and closure of all public and education facilities (early childhood education centres and schools etc.).

^d^ Patial border relaxation 1: quarantine‐free travel with Australia during 19 April 2021 to 22 July 2021.

^e^ Patial border relaxation 2: progressive border relaxation between 28 Feburary to 31 July 2022. Introduction of quarantine‐free travel initially for vaccinated New Zealanders from Australia on 28 Feburary 2022 and for the same groups from the rest of the world on 13 March2022, then for vaccinated Australians from 13 April 2022 and vaccinated travellers from NZ's visa‐waiver countries from 2 May 2022 onwards.

## 4 DISCUSSION

NZ, a southern hemisphere island country with a temperate climate, has a well‐established pattern of influenza and RSV circulation with annual peak incidence usually in the winter months from June to September.^26^ NZ's absence of community transmission of influenza and RSV after May 2020, largely due to COVID‐19 elimination measures,^5^ provided a unique opportunity to describe the impact of border restrictions and relaxations on these viral infections for the subsequent 2 years, because overseas travellers became their only source of re‐introduction. Comprehensive surveillance from hospital, GPs and community cohorts showed that initial stringent PHSMs (lockdown, school closure and border closure) were able to remove influenza and RSV from the NZ community, and the subsequent tight border restrictions in absence of other stringent PHSMs appeared to be effective at keeping them out. Re‐introduction of RSV and influenza into NZ were temporally associated with partial border relaxations in 2021 and 2022, respectively. However, border restrictions did not have much impact on non‐enveloped viruses such as rhinovirus and some of the enveloped viruses such as PIV1, probably due to their suppressed (but not eliminated) transmission within NZ during the initial stringent PHSMs or ineffectiveness of border closure.

The WHO's pandemic influenza intervention guidance does not recommend border restrictions when pandemic influenza emerges in human populations because these measures have been considered ineffective and impractical.^27^ However, the knowledge base used in developing WHO guidance for influenza pandemic prevention consists primarily of historical observations and modelling studies that provide generally poor quality of evidence/data.^28^, ^29^, ^30^ NZ's high‐quality data generated during the COVID‐19 pandemic questions some of the rationales underpinning the WHO guidance. Our conclusions drawn from multiple NZ surveillance streams on the effectiveness of border restrictions in preventing influenza transmission are consistent with those reported from other countries including Australia,^14^, ^31^, ^32^, ^33^ Hong Kong,^11^ Chile^34^ and South Africa.^35^ Therefore, we suggest that it is important to re‐evaluate the role of border restrictions (and PHSMs generally) in delaying, mitigating or even potentially eliminating influenza pandemics. Although such measures are associated with significant negative impacts on society, the potential beneficial effects of delaying respiratory viral transmission can provide the time needed for developing, producing, and distributing vaccines and therapeutics that can prevent death and disease. New knowledge from this assessment may inform better preparedness for future influenza pandemics and other severe respiratory viral threats.

While NZ's RSV absence from May 2020 continued during border closure and easing of other stringent PHSMs (lockdown and childcare/school closure), community transmission of RSV returned from April 2021 soon after border relaxation with Australia. NZ's situation is different from Australia where a peak of RSV cases was observed from September 2020 followed easing of restrictions on gatherings and school re‐openings but preceding the relaxation of border restrictions. This suggested that, unlike NZ, the initial PHSMs did not effectively remove RSV from the Australia community, or alternatively incomplete border closures.^36^ One important difference between Australia and NZ's pandemic restrictions (relevant for RSV) is that Australia allowed childcare centres to mostly remain open during pandemic restrictions, providing opportunities for maintaining RSV circulation.^24^ Indeed, during the 2020/21 RSV season in Europe, where overall RSV activity was very low, the only countries with major RSV outbreaks were those with policies to keep primary school and childcare centres open.^37^ A detailed analysis of temporal trends in RSV infections around the time of implementation and lifting of specific interventions (mask mandates, school closures, travel restrictions etc.) can provide valuable insights into effective strategies to prevent/mitigate future epidemics of RSV in each local context.

Not all respiratory viruses were impacted by border restrictions, especially those non‐enveloped respiratory viruses (rhinovirus, enterovirus and adenovirus). Rhinovirus persisted throughout border restrictions in NZ. Rhinovirus' non‐enveloped nature,^38^ persistence in environment^39^ and high prevalence in population^40^ may account for it being less affected by PHSMs including border restrictions. Interestingly, PIV1 (an enveloped virus) was also less affected by NZ's border restrictions because we observed a rapid increase of PIV1 incidence from November 2020 to January 2021 during border closure. Unlike influenza and RSV, prolonged shedding of low levels of PIV has been documented in normal asymptomatic healthy adults,^41^ children^42^ and immunocompromised persons.^43^ The prolonged shedding may account for PIV1 local transmission being suppressed (not eliminated) by the short period (5 weeks) of initial stringent PHSMs implemented in 2020, and then returning after easing of these restrictions. Alternatively, prolonged PIV1 shedding may account for ineffectiveness of border closure as the length of the MIQ stay is <14 days for each traveller. This would provide opportunities for PIV1‐infected travellers to continue shedding the virus after the release from MIQ, seeding the virus into the NZ community. Whole genome sequencing may help in distinguishing these two possible scenarios.

The strengths of our study are: (1) NZ's unique setting allowed us to disentangle the effect of the stringent border restrictions from other stringent measures (stay‐at‐home orders, school closure etc.) on influenza, RSV and other common respiratory viral infections. The brief border opening with Australia created seeding opportunity for some of the respiratory viruses such as RSV, which contributed to novel data and knowledge on impact and temporal association of the border restrictions and virus importation. (2) The active surveillance from hospital, GP, community cohorts and passive laboratory surveillance provided concordance data on the impact of the border restrictions on these respiratory viral infections with a range of disease spectrums. (3) The hospital, GP and community cohort surveillance had well‐defined denominators for calculating population‐based incidence rates. (4) Our study utilised a large dataset that was collected prospectively, including nine of the most prevalent non‐COVID‐19 respiratory viruses and up to 8 years of weekly testing data (from 2015 to 2022).

The limitations of our study are: (1) all our surveillance systems were triggered when patients experienced acute respiratory illnesses with subsequent swabbing and testing. We had no real‐time routine surveillance for swabbing asymptomatic individuals who may have influenza/RSV infections. (2) The number of laboratory detections of influenza/RSV/other respiratory viruses for hospital patients during routine clinical practice is influenced by testing technology, instruments, reagents, priorities, demands and human resources during the COVID‐19 pandemic. Additionally, these samples ordered by clinicians based on clinical judgement may result in selection bias. Furthermore, this surveillance system only reports positive viral detections; thus, there are no data on proportion of positives among tested samples. (3) The COVID‐19 pandemic might interrupt the usual patient flow for sentinel GPs. This might result in lower consultations and under‐reporting for ILI rates during 2020–2022.

In conclusion, NZ's unusual experience of influenza and RSV absence from May 2020, due to COVID‐19 pandemic elimination measures, allowed us to examine the impact of border restrictions and relaxations over the subsequent 2 years on influenza, RSV and other respiratory viral infections. Our findings showed that total border closure to most non‐residents and mandatory government‐MIQ on arrival for those allowed to enter appeared to be effective in preventing influenza and RSV spread into NZ. Border relaxation through quarantine‐free travel was quickly followed by importation of RSV and influenza into NZ. Border restrictions did not have much impact on other respiratory viruses such as rhinovirus and parainfluenza virus type‐1. Our data provide important insights into the role of border restrictions in managing future pandemic threats from influenza and other severe respiratory viruses. Our findings show that elimination provides a feasible alternative to mitigation, which has been a dominant pandemic strategy to date.^44^ Our results also provide insights into the global circulation pattern and epidemiology of human respiratory pathogens before, during and after the COVID‐19 pandemic. These ‘real‐world’ data can facilitate future modelling studies by providing the precision and accuracy of predictions for the timing and severity of seasonal influenza, RSV and other respiratory viral outbreaks.

## AUTHOR CONTRIBUTIONS

All authors meet the International Committee of Medical Journal Editors criteria for authorship. Q. Sue Huang, Nikki Turner, Peter McIntyre, Nayyereh Aminisani, Tony Dowell, Adrian Trenholme, Cass Byrnes, Michelle Balm, Christine McIntosh, Sarah Jefferies, Cameron C. Grant, Annette Nesdale, Hazel C. Dobinson, Priscilla Campbell‐Stokes, Karen Daniells, Andrea McNeill, Tomasz Kiedrzynski, Sally Roberts, Colin McArthur, Conroy Wong, Michael G. Baker, Amanda Kvalsvig, Marc‐Alain Widdowson, Paul G. Thomas and Richard J. Webby designed and operationalised the SARI, ILI and/or SHIVERS‐II, ‐III, and ‐IV cohort platforms. Lauren Jelley, Jemma Geoghegan, Joep de Ligt, Chor Ee Tan, Xiaoyun Ren, Klarysse Berquist, Meaghan O'Neill, Maritza Marull, Chang Yu, Alicia Stanley, Susan Taylor, Shirley Lawrence, Koen Van Der Werff, Gary McAuliffe, Hanna Antoszewska, Meik Dilcher, Jennifer Fahey, Anja Werno, Juliet Elvy, Jenny Grant, Michael Addidle, Nicolas Zacchi, Chris Mansell, Tim Wood and Andrew Anglemyer provided the testing and reporting. Ruth Seeds, Tineke Jennings, Megan Rensburg, Jort Cueto, Ernest Caballero, Joshma John and Emmanuel Penghulan did the clinical data and samples collection and reporting and ensured operations. Tim Wood and Q. Sue Huang did the data analysis. Q. Sue Huang wrote the first draft of the manuscript. All authors contributed to the interpretation of the results, revision of the manuscript critically for intellectual content and have given final approval of the version to be published.

## CONFLICT OF INTEREST STATEMENT

The authors declare that they have no competing interests.

### PEER REVIEW

The peer review history for this article is available at https://www.webofscience.com/api/gateway/wos/peer-review/10.1111/irv.13247.

## ETHICS STATEMENT

Ethical approval was obtained for the SHIVERS (including SARI and ILI/ARI surveillance), SHIVERS‐II, ‐III and ‐IV cohort studies and SHIVERS‐V surveillance from the NZ Northern A Health and Disability Ethics Committee (NTX/11/11/102). The laboratory‐based respiratory virus surveillance data are part of public health surveillance in NZ. This surveillance is conducted in accordance with the Public Health Act, and thus, ethics committee approval was not needed for collection or use of these data.

## Supporting information


**Figure S1** Laboratory‐based positive parainfluenza virus (PIV) types 1–3 reported to the Institute of Environmental Science and Research (ESR) during 2003–2022
**Table S1** Percentage of tested samples before, during and after border restrictions, 2019–2022

## Data Availability

The authors welcome queries about possible collaborations and requests for access to the data. Data including line list participant data and a data dictionary defining each variable will be shared after approval of a proposal and with a signed data access agreement. Researchers interested in more details about this study should contact the principal investigator and corresponding author, Q. Sue Huang (sue.huang@esr.cri.nz).
