# Supplementary material for: Impact of the COVID‐19 related border restrictions on influenza and other common respiratory viral infections in New Zealand
Source: Influenza Other Respir Viruses. 2024 Feb 13;18(2):e13247. doi: 10.1111/irv.13247 (PMC10864123; doi:10.1111/irv.13247)
Supplement: Supplementary file 1 — Figure S1 Laboratory‐based positive parainfluenza virus (PIV) types 1–3 reported to the Institute of Environmental Science and Research (ESR) during 2003–2022 Table S1 Percentage of tested samples before, during and after border restrictions, 2019–2022 [file IRV-18-e13247-s001.docx]

**Impact of the COVID-19 related border restrictions on influenza and other common respiratory viral infections in New Zealand**

**Huang et al. Supplementary Information Appendix**

**Supplementary Figure.** Laboratory-based positive parainfluenza virus (PIV) types 1-3 reported to the Institute of Environmental Science and Research (ESR) during 2003-2022

**Supplementary Table.** Percentage of tested samples before, during and after border restrictions, 2019-2022
